# KRAS in NSCLC: State of the Art and Future Perspectives

**DOI:** 10.3390/cancers14215430

**Published:** 2022-11-04

**Authors:** Priscilla Cascetta, Arianna Marinello, Chiara Lazzari, Vanesa Gregorc, David Planchard, Roberto Bianco, Nicola Normanno, Alessandro Morabito

**Affiliations:** 1 Department of Medical Oncology, Gustave Roussy Cancer Campus, 114 rue Edouard Vaillant, 94850 Villejuif, France; 2 Department of Medical Oncology, IRCCS Humanitas Research Hospital, Via Manzoni 56, 20089 Rozzano, Italy; 3 Department of Medical Oncology, Candiolo Cancer Institute, FPO-IRCCS, Candiolo, 10060 Turin, Italy; 4 Department of Clinical Medicine and Surgery, Oncology Division, University of Naples Federico II, Via Sergio Pansini 5, 80131 Naples, Italy; 5 Cellular Biology and Biotherapy, Istituto Nazionale Tumori, IRCCS, Fondazione G. Pascale, Via Mariano Semmola 53, 80131 Naples, Italy; 6 Thoracic Medical Oncology, Istituto Nazionale Tumori, IRCCS, Fondazione G. Pascale, Via Mariano Semmola 53, 80131 Naples, Italy

**Keywords:** KRAS, NSCLC, lung cancer, adagrasib, MRTX849, sotorasib, AMG 510, immune-checkpoint inhibitors, acquired resistance

## Abstract

**Simple Summary:**

Rat sarcoma virus (RAS) GTP-ase proteins represent a key element in cellular proliferation, growth, and differentiation. Three different isoforms of RAS proteins have been identified to date (KRAS, NRAS, HRAS) and mutations in KRAS are frequently found in human cancers, among which the Non-Small Cell Lung Cancer (NSCLC). In this review we assess molecular, prognostic, and clinico-pathological characteristics of KRAS mutations in NSCLC patients. Next, we present current therapeutic strategies for KRAS mutant NSCLC patients and mechanisms of acquired resistance identified to date. We then focus on the role of immune-checkpoint inhibitors in KRAS mutant NSCLC patients. Finally, we will overview ongoing trials and future needs for this subpopulation cohort.

**Abstract:**

In NSCLC, KRAS mutations occur in up to 30% of all cases, most frequently at codon 12 and 13. KRAS mutations have been linked to adenocarcinoma histology, positive smoking history, and Caucasian ethnicity, although differences have been described across KRAS mutational variants subtypes. KRAS mutations often concur with other molecular alterations, notably TP53, STK11, and KEAP1, which could play an important role in treatment efficacy and patient outcomes. For many years, KRAS mutations have been considered undruggable mainly due to a high toxicity profile and low specificity of compounds. Sotorasib and adagrasib are novel KRAS inhibitors that recently gained FDA approval for pre-treated KRAS mutant NSCLC patients, and other molecules such as GDC-6036 are currently being investigated with promising results. Despite their approval, the efficacy of these drugs is lower than expected and progression among responders has been reported. Mechanisms of acquired resistance to anti-KRAS molecules typically involves either on target secondary mutations (e.g., G12, G13, Q61H, R68S, H95, Y96C, V8L) or off-target alterations. Ongoing trials are currently evaluating strategies for implementing efficacy and overcoming acquired resistance to these compounds. Finally, the efficacy of immune-checkpoint inhibitors still needs to be completely assessed and responses to anti-PD-1/PD-L1 agents may strongly depend on concomitant mutations.

## 1. Introduction

With almost 1.8 million deaths per year, lung cancer remains the leading cause of cancer-related death worldwide. Non-small cell lung cancer (NSCLC) accounts for 85% of all diagnosed cases, with adenocarcinoma being the most common histological subtype [1,2]. Progress in molecular characterization and development of new targeted therapies have changed the treatment scenario of NSCLC in recent years. So far, molecular alterations thought to be undruggable for many years, such as KRAS mutations, may now benefit from targeted agents. 

KRAS (Kirsten Rat sarcoma virus) proteins are small GTP-ases belonging to the wider family of RAS proteins. The KRAS gene is located in the short arm of chromosome 12 (12 p) and its transcripts may further undergo an alternative splicing process, resulting in two distinct proteins of approximately 21 KDa each: KRAS 4A and KRAS 4B [3]. Even though differences in physiological expression have been reported in some preclinical data [4], little is known about the real impact of these alternatively spliced variants in NSCLC. 

Structurally, all RAS isoforms have a highly conserved catalytic region, implied in GTP bounding and hydrolysis. Differences across RAS isoforms are mainly founded at the C-terminal hyper variable region (HVR) domain, responsible for post-transcriptional modifications and interaction with the phospholipidic membrane bilayer [5,6]. 

In physiological conditions, KRAS proteins are key elements in transducing signaling from activated receptor tyrosine kinase (RTK) to downstream intracellular pathways. Indeed, intracellular KRAS proteins may present in two distinct functional forms: KRAS-GTP and KRAS-GDP, which correspond to “active” and “inactive” states, respectively. Transition from the inactive state (KRAS-GDP) to active state (KRAS-GTP) is usually mediated by the guanine nucleotide exchange factors (GEFs) via the releasing of guanosine diphosphate (GDP) and the subsequent binding of guanosine triphosphate (GTP). Many GEFs proteins have been identified to date, with the GEF Son of Sevenless (SOS1) playing a crucial role in KRAS activation. Of note, this process determines key structural modification in KRAS proteins mainly involving the switch pockets I and II, which serve as the binding interface for effector proteins [7,8]. However, the transition from the inactive to active form is complex and usually requires other proteins (e.g., the adaptor protein Grb2 and the SOS1/Grb2 recruiter SHP-2) which directly interact with upstream phosphorylated RTKs [9]. Contrarily, transition from the KRAS-GTP to KRAS-GDP binding state is facilitated by GTPase activating proteins (GAPs) such as NF1, which accelerate the hydrolysis of GTP to GDP [10]. 

**Figure 1:**
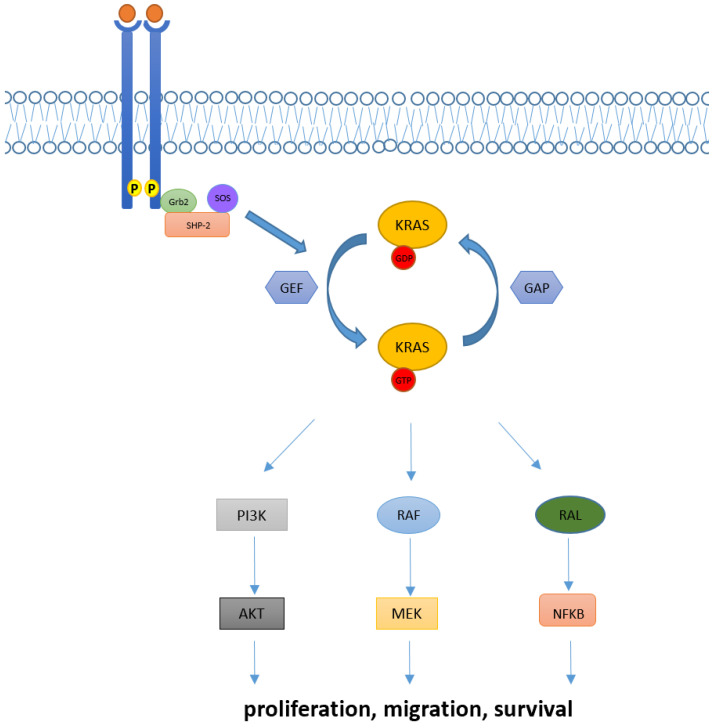
KRAS activation and its downstream pathway. KRAS proteins are key elements in transducing extracellular signaling to downstream pathways. Intracellular KRAS proteins may present in two functional forms, KRAS-GTP and KRAS-GDP, which correspond to their active and inactive state, respectively. Transition from the KRAS-GDP to KRAS-GTP binding state is usually mediated by GEFs proteins and occurs via other adaptor and recruiter proteins, such as Grb2 and SHP-2. Conversely, transition from KRAS-GDP to KRAS-GTP usually requires GAPs proteins. Downstream signaling of KRAS normally includes RAF-MEK-ERK, PI3K/AKT and RAL pathways, implied in the regulation of cellular proliferation, migration, and death.

Downstream pathways of KRAS normally include the RAF-MEK-ERK mitogen-activated protein kinase (MAPK) pathway, the phosphatidylinositol 3-kinase (PI3K)-AKT-mTOR, and the RAS-like (RAL) pathways [5], whose deregulations have been linked with abnormal cellular proliferation, migration, and death (Figure 1).

The current review provides an overview of the biology and clinic-pathological characteristics of KRAS mutant NSCLC patients. Moreover, we discuss therapeutic strategies that have been explored in these subgroups of patients and the clinical data leading to the approval of sotorasib and adagrasib, the first compounds that efficiently inhibit KRAS G12C mutation. We then focus on acquired resistance mechanisms evidenced to date and we further assess the role of immune checkpoint inhibitors in KRAS mutant patients. Finally, we look into ongoing trials and new KRAS inhibitors, which are currently being tested. 

### 1.1. Techniques for Detection of KRAS Mutations 

The assessment of the KRAS status in patients affected by colorectal cancer and NSCLC is mandatory for a correct therapeutical management [11]. The main techniques allowing KRAS point mutations are polymerase chain reaction (PCR) based methods, such as high-resolution melting and real time RCP (RT.-PCR), and next generation sequencing (NGS) [12,13,14]. RT-PCR allows the highly sensitive qualitative detection and identification of a fixed number of mutations in specific areas of the gene [13]. Highly verified assays, such as Cobas Mutation Test V2, reaches a 100% level of accuracy in tissue samples, analyzing exons 2, 3, and 4 of the KRAS gene [15]. NGS technologies, being DNA-based or RNA-based, enable the simultaneous analysis of a high variety of genes and loci, at the price of reducing sensitivity, when compared to PCR [16]. With the advent of new molecular targets, an exhaustive characterization of lung cancer patients has become essential, making NGS an extremely valuable tool [17,18,19,20].

The sensitivity of KRAS mutation detection has been evaluated and compared in various types of specimens, such as tissue, plasma, and other body fluids, finding a high rate of concordance between tissue and liquid biopsy both for PCR and NGS [21,22]. The use of liquid biopsy is of great relevance for the management of advanced NSCLC, enabling the identification of KRAS mutations at the baseline, but also to picture disease heterogeneity, to evaluate response to treatment in time and to identify molecular mechanisms of resistance to targeted molecules [22,23,24]. Overall, tissue-based testing remains the preferred test for most cancer patients, although liquid biopsy may be chosen when faster results will be clinically important, or when obtaining tissue specimens is not possible or inappropriate [23] However, it is important to point out that reimbursement for broad coverage is still limited [25].

### 1.2. KRAS in NSCLC: Different Alterations and Patients’ Characteristics 

In NSCLC, KRAS mutations are founded in up to 30% of all cases and most frequently involve the codon 12 (90% of cases), while less common mutations are observed in codon 13 (2–6%) and 61 (1%) [26,27]. Within codon 12 mutations, the one resulting in the substitution of glycine with cysteine (KRAS G12C) represents the most prevalent KRAS alteration in NSCLC (40% of all KRAS mutant cases), followed by substitution of glycine with valine (KRAS G12V, 21%), and substitution of glycine with aspartic acid (KRAS G12D, 17%). Indeed, other point mutation in codon 12 such as G12A/R/S are rare [26,27,28]. Functionally, mutations at codon 12 and 13 result in reduced GAP-mediated hydrolysis via allosteric bloc that hinders the GTP-ase site of KRAS, whereas alterations in codon 61 abolish both intrinsic and GAP-mediated GTP hydrolysis of KRAS [29]. Of note, KRAS mutant variants can differently activate their downstream pathway. Indeed, the hydrophobic G12C and G12V preferentially activate the RAL pathway, while the hydrophilic G12D mainly acts via PI3K-AKT signaling. KRAS G12C weakens the interaction with PI3K, and the bulky aspartic acid in the G12D prevents the formation of the binding with RelA/RelB [30]. Besides NSCLC, KRAS mutations are also frequent in other tumor types, such as pancreatic and colorectal adenocarcinoma (88% and 50% of all cases, respectively) [28,31], with KRAS G12D mutations being the most common alteration among these histotypes (32% and 46% of all cases, respectively) [32].

In lung cancer patients, KRAS mutations have been associated with Caucasian ethnicity (Caucasian vs. Asian population: 26% vs. 11%), female sex (female vs. males: 31.35 vs. 23.7%, *p* < 0.0001), adenocarcinoma histology (adenocarcinoma vs. squamous cell carcinoma: 37.2% vs. 4.4%), and a positive smoking history (smokers vs. non-smokers: 30% vs. 11%) [26,28]. However, these characteristics may strongly depend on single mutant KRAS variants. As such, KRAS G12C/G12A/G12V/G13C mutations are more frequently found in current or former smokers, while prevalence of KRAS G12D/G12S/G13D mutations is higher among non-smokers [26,33]. Furthermore, the median age for KRAS G12C patients seems to be significantly lower than that observed for other KRAS mutants (KRAS G12C vs. other KRAS-MUT: 63.1 vs. 65.9 years, *p* = 0.0092) [34], although this evidence has not been further confirmed in other reports [26]. Finally, the metastatic pattern could also slightly differ across single KRAS mutant variants, since KRAS G12C mutants are more likely associated with lung metastasis (KRAS G12C vs. non–G12C: 38% vs. 21%; *p* = 0.043) and less associated with either pleural metastasis or lymphangitic carcinomatosis (KRAS G12C vs. non–G12C: 4% vs. 39%, *p* = 0.0001) [35]. Of note, KRAS mutant patients do not have a higher brain tropism than those without KRAS alterations, and the same incidence of brain metastasis has been observed across different KRAS mutant variants (KRAS mut vs. KRAS wild-type: 33% vs. 40%, *p* = 0.17; KRAS G12C vs. KRAS other: 40% vs. 41%, *p* = 0.74) [36]. 

### 1.3. Predictive and Prognostic Role of KRAS Mutations in NSCLC Patients

Multiple studies have evaluated the predictive and prognostic role of KRAS mutations in NSCLC. 

In an early setting, different reports have demonstrated similar overall survival (OS) and disease-free survival (DFS) in KRAS mutants versus wild type patients (OS HR = 1.17, DFS HR = 1.04) [37,38]. However, these results were not further confirmed and other reports eventually demonstrated a worse OS and DFS for KRAS mutant patients (DFS *p* = 0.038, OS HR = 1.30; *p* = 0.002) [37]. 

It has been asked if the prognostic and predictive significance of KRAS mutation could depend on specific variants. Indeed, Finn et al. recently demonstrated a negative prognostic impact for KRAS G12C completely resected patients compared to both the KRAS wild type and other mutant variants (OS KRAS G12C versus other KRAS HR = 1.39, *p* = 0.031; KRAS G12C versus no KRAS HR = 1.32, *p* = 0.028) [34]. Contrarily, no prognostic nor predictive value for codon 12 mutations has been identified in a pooled meta-analysis conducted in early stage disease, while codon 13 mutations have been associated with a possible deleterious effect to adjuvant chemotherapy [37]. 

In an advanced setting, a meta-analysis of 43 selected studies demonstrated an impaired OS and progression-free survival (PFS) for KRAS mutants (OS HR = 1.71; 95% CI [1.07, 2.84]; PFS HR = 1.18; 95% CI [1.02, 1.36]). However, these results were only observed when considering observational studies and not further confirmed in randomized clinical trials (OS HR = 1.10; 95% CI [0.88–1.38]; PFS HR = 1.03; 95% CI [0.80–1.33]) [39]. Likewise, discordant results about the prognostic and predictive role of KRAS mutations have been reported in the advanced setting [27,40,41]. Furthermore, no differences in OS have been observed across different KRAS mutations, although a better response to taxanes has been reported for KRAS G12V mutants [33,42]. 

In conclusion, the prognostic and predictive role of KRAS mutations in NSCLC is still a matter of debate and discordant results provided so far need further assessment. 

### 1.4. Concurrent Molecular Alterations in KRAS Positive NSCLC Patients 

Concurrent molecular alterations are often found in KRAS mutant NSCLC patients. In the largest cohort available to date, Riely et al. described a median of seven concomitant mutations in KRAS positive NSCLC patients, the most frequent being TP53 (39%), STK11 (30%), KEAP1 (24%), RBM10 (15%), and PTPRD (15%). Curiously, those harboring concomitant mutations in either STK11 or KEAP1 had a shorter OS (STK11: HR = 2, *p* = 0.006; KEAP1: HR = 2.8, *p* < 0.001) and the negative prognostic impact of STK11 and KEAP1 co-alterations has been further confirmed in multiple studies [43,44]. Of note, neither the TP53 status nor number of concurrent mutations affected OS [45]. In another retrospective study, Passaro et al. demonstrated that 77% of patients with KRAS mutations had a concurrent molecular alteration. Interestingly, the percentage of concomitant alterations did not differ between G12C and non G-12C KRAS mutants. Again, the most frequently reported co-alteration was TP53 (23%) followed by STK11 (15%). Importantly, alterations in CDK2/4 and receptor tyrosine-kinase (RTK) have also been described in KRAS mutant cases [46]. 

Importantly, concurrent alterations might affect the tumor microenvironment with consequent alterations in the immunogenic phenotype. As such, Skoulidis et al. demonstrated a lower immunogenic infiltrate in STK11 positive tumors, while the opposite was seen for concomitant TP53-mutated tumors [47]. 

It has also been demonstrated that co-mutations could differentially activate downstream pathways. Indeed, engineered mice harboring KRAS G12D alone or with concomitant TP53 or STK11 mutations, respectively, hyper-activated MEK/ERK or AKT/SRC pathways [48]. 

Concurrent alterations with other key oncogenic drivers have also been described. Of note, KRAS mutations are found in approximately 3% of MET ex14 alterations [49,50], and a few cases of concomitant KRAS/ALK and KRAS/ROS1 alterations have been reported [51,52]. Interestingly, KRAS mutations have been found as mechanisms of resistance to the first, second, and third generation of EGFR TKI [53,54,55,56].

## 2. Targeted Therapy in KRAS Mutant NSCLC

**Figure 2:**
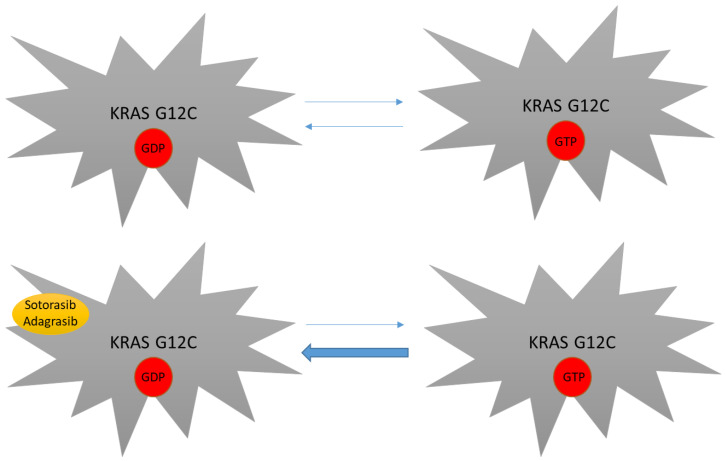
Mechanism of action of novel KRAS inhibitors. KRAS G12C mutants still depend on their GTP binding state in order to be fully active. By covalently binding the switch pocket II, sotorasib and adagrasib reduce KRAS G12C-GDP availability, thus impairing transition to the KRAS-GTP active form.

**Figure 3:**
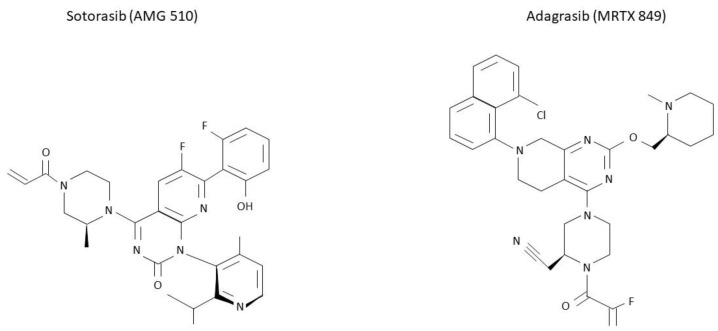
Molecular structure of sotorasib and adagrasib. KRAS G12C inhibitors sotorasib and adagrasib have been recently approved for a similar setting. However, differences in their molecular structure are here presented.

As mentioned above, KRAS mutations have been considered undruggable for many years. Difficulties in targeting this protein were mainly linked to its smooth surface coupled with its high affinity to GTP, which made the targeting of the GDP/GTP binding site very challenging [29]. The subsequent discovery of the switch pocket II of KRAS protein represented a breakthrough in the treatment of KRAS mutations and molecules that specifically bind cysteine residues of KRAS mutant proteins were further developed. Because this binding preferentially occurs in the RAS-GDP form, KRAS G12C inhibitors mainly act by trapping KRAS-GDP proteins and consequently by impairing their transition to the KRAS-GTP active state [29,57,58,59] (Figure 2). Even though different molecules have been developed to date, sotorasib and adagrasib are the two having received regulatory approval to date (Figure 3). 

### 2.1. Sotorasib (AMG 510)

Sotorasib (AMG 510) was one of the first molecules entered in clinical trials. In the single-arm, phase I-II CodeBreaK100 trial, the efficacy and safety of sotorasib were evaluated in different types of KRAS G12C mutant cancers [60,61]. The phase I trial enrolled 129 patients, 59 of whom affected by advanced NSCLC [60]. The primary endpoint was safety. KRAS G12C-mutated NSCLC patients were heavily pre-treated, with a median of three (range 0–11) previous lines of anticancer therapy for metastatic disease. Treatment-related adverse events (AEs) of any grade were observed in 56.6% of patients. Grade 3 treatment-related AEs included an increase in the alanine aminotransferase (ALT) level (4.7%), diarrhea (3.9%), anemia (3.1%), and an increase in the aspartate aminotransferase (AST) level (2.3%). AEs for any cause were reported in 96.9% of cases, mainly diarrhea (29.5%), fatigue (23.3%), and nausea (20.9%). Grade 3 or higher of all related AEs were reported in 68 patients (52.7%), consisting of anemia, liver enzymes alteration, and diarrhea. No dose-limiting toxic effects nor toxic deaths were observed. The dose of 960 mg administered daily was identified as the dose for the expansion cohort. As for efficacy in NSCLC, an overall response rate (ORR) of 32.2% and a disease control rate (DCR) of 88.1% were observed, with 71.2% of patients showing tumor-shrinkage at the first assessment. After a median follow-up of 11.7 months (range 4.8 to 21.2), median time to response (mTTR) was 1.4 months (range 1.1 to 9.5), median duration of response (mDoR) was 10.9 months (range 1.1+ to 13.6), median PFS (mPFS) was 6.3 months (range 0.0+ to 14.9). 

Given the aforementioned results, the phase II of this trial started [61] (Table 1). This second part of the trial enrolled 126 patients with NSCLC harboring KRAS G12C mutation, who had progressed after anti-PD-1/PD-L1 molecules and platinum-based chemotherapy alone or combined. Patients with stable brain metastases were allowed. The primary endpoint was ORR, key secondary endpoints were DoR, DCR, TTR, PFS and OS. Sotorasib was administered at the identified dose of 960 mg orally once daily. Median number of systemic therapy was 2, patients receiving more than 3 previous lines were excluded. After a median follow up of 15.3 months (range 1.1–18.4+), 37.1% of patients obtained complete response (CR) or partial response (PR) (4 CR, 3.2%, 42 PR, 33.9%), and DCR was 80.6%. Responders had rapid and durable benefit (mTTR 1.4 months, range 1.2–10.1; mDoR 11.1 months, range 6.9-NE). mPFS was 6.8 months (95% CI, range 5.1 to 8.2), mOS was 12.5 months (95% CI, range 10.0 to NE). As for safety, treatment-related AEs were consistent with previous data and observed in overall 69.8% of patients. Again, most common AEs were diarrhea (31.7%), nausea (19%), fatigue (11%), and liver enzyme alteration. (15.1%). Grade 3 AEs were observed in 19.8% of patients and grade 4 in 1 patient (0.8%, consisting in pneumonitis and dyspnea); no toxic deaths were reported. Treatment modification (interruption, dose reduction, or both) occurred in 22.2% of patients and discontinuation of experimental therapy was reported in 7.1% of cases. 

In terms of concurrent alterations, responses to sotorasib were evaluated in those harboring co-mutation in either STK11 or KEAP1 (STK11+/KEAP 1− or STK11−/KEAP1+) as well as in those with concomitant STK11 and KEAP1 co-alterations (STK11+/KEAP1+). Responses were higher in the STK11+/KEAP1− patients (50%), while worst outcomes were seen in STK11−/KEAP1+ mutants (14%). Responses for STK11+/KEAP1+ patients were placed in the middle (23%). Of note, RR was not significantly different in TP53-mutated versus wild-type patients (RR in TP53-mutated vs. wild-type: 39% vs. 40%), while KEAP1− mutated patients had detrimental results compared to KEAP1 wild-type after sotorasib exposure (RR in KEAP1-mutated vs. wild-type: 20% vs. 44%). 

Interestingly, a post-hoc analysis demonstrated intracranial efficacy of sotorasib. Of note, among 40 patients with evaluable brain metastasis at baseline, ORR was 25% and DCR was 87.5% [62]. 

Preliminary results of the CodeBreak 200 trial were presented at ESMO 2022. This was a global, open-label, randomized phase III trial evaluating the efficacy of sotorasib versus docetaxel in KRAS mutant NSCLC patients who progressed after prior platinum-based chemotherapy and a checkpoint inhibitor. The primary endpoint was PFS, key secondary endpoints included OS, DCR, ORR. After study initiation and per regulatory guidance, initial planned enrolment was reduced and the crossover to sotorasib was allowed in docetaxel progressing patients. At a median follow-up of 17.7 months, sotorasib demonstrated a longer PFS compared to docetaxel (HR= 0.66 [95% CI: 0.51, 0.86], *p* = 0.002), with a 1-y reported PFS of 24.8% versus 10.1% in sotorasib and docetaxel arm, respectively. Among secondary endpoints, ORR and DCR were in favor of sotorasib (ORR sotorasib vs. docetaxel: 28.1% vs. 13.2%; *p* < 0.001; DCR sotorasib vs. docetaxel: 82.5% vs. 60.3%), while no differences in terms of OS were evidenced. In terms of safety, sotorasib was better tolerated than docetaxel and, consistently with the aforementioned results, most frequently reported Grade ≥3 secondary effects of sotorasib arm were diarrhea (33.7%), nausea (14.2%), fatigue (6.5%), hepatic enzyme alterations (ALT/AST increase in 10.1% of cases each) [63]. 

Based on these results, sotorasib received accelerated FDA approval in May 2021 for the treatment of patients who had received at least one prior line of systemic therapy [64]. 

### 2.2. Adagrasib (MRTX849)

Adagrasib (MRTX849) was the second KRAS G12C inhibitor that was entered in clinic trials [59]. The phase I/Ib dose-finding component of the first-in-human KRYSTAL-1 trial evaluated the safety, pharmacokinetics (PK), and clinical activity of adagrasib in KRAS G12C mutant advanced solid tumors [65]. In this trial, a total of 25 mutant patients were enrolled and 18 (72%) of them were affected by NSCLC. After PK and safety assessment, 600 mg twice a day was chosen as the recommended phase II dose (RP2D). Overall, 23 patients (92.0%) experienced treatment-related AEs and most frequently nausea (76.0%), diarrhea (72.0.0%), vomiting (48.0%), and fatigue (40.0%). Severe AEs (G ≥ 3) were reported in 36% of patients and the most common consisted in fatigue (15.0%). Additionally, one grade 5 pneumonitis occurred in one patient with underlying radiation and underlying pneumonitis. Furthermore, nausea (25.0%), diarrhea (20%), vomiting (20%) and fatigue (20%) were the main AEs responsible for treatment interruption or dose reduction at 600 mg twice daily. Among 15 evaluable patients with NSCLC treated with adagrasib at RP2D, ORR was 53.3%. After a median follow-up time of 19.6 months, mDOR was 16.4 months (95% CI, 3.1 to NE; range 2.8–16.9), while mPFS was 11.1 months (95% CI, range 2.6 to NE).

Given these results, the expansion phase of KRISTAL-1 trial was conducted [66] (Table 1). This second part of the trial enrolled a total of 116 KRAS mutant NSCLC patients. Patients enrolled were pre-treated with two median lines of systemic therapies and 14 (12.1%) of them received at least four previous systemic regimens. Globally, 98.3% of included patients received chemotherapy and immunotherapy as previous lines of therapy. Among 112 patients with measurable disease at the baseline, 48 (42.9%) showed an objective response and DCR was observed in 79.5% of cases. In particular, one patient obtained a CR (0.9%), 47 (42%) had PR, and 41 had (36.6%) stable disease (SD) as their best response. mDoR was 8.5 months (95% CI, 6.2–13.8) and mPFS was 6.5 months (95% CI, range 4.7–8.4). At the last update, after a median follow-up of 15.6 months, mOS was 12.6 months (95% CI, range 9.2–19.2). Considering patients with stable, previously treated CNS metastases, an intracranial confirmed response rate of 33.3% (95% CI, range 8.0–51.8) was reported. Further analysis of patients carrying co-occurring alterations in STK11, KEAP1, or both, confirmed a detrimental effect in those harboring KEAP1 co-mutations (ORR 14.3% in STK11-/KEAP1+). As for safety, treatment-related AEs occurred in 97.4% of patients, mainly grade 1 or 2, while 44.8% of them were G3 or higher. Most common treatment-related AEs were diarrhea (62.9%), nausea (62.1%), vomiting (47.4%), fatigue (40.5%), an increased alanine aminotransferase (ALT) level (27.6%), an increased blood creatinine level (25.9%), and an increased aspartate aminotransferase (AST) level (25.0%). Furthermore, two fatal events were reported (one cardiac failure and one pulmonary hemorrhage) and drug discontinuation related to AEs was observed in 8 (6.9%) patients, while AEs led to dose reduction and dose interruption in 51.7% and 61.2% of all cases, respectively.

In terms of intracranial responses, ORR of adagrasib was 33.3% and among patients with brain metastasis at baseline the mDoR was 11.2 months. The efficacy of adagrasib is currently being investigated in a phase 3 trial versus docetaxel in pretreated patients (KRISTAL-12, NCT04685135) [67].

Based on these results, adagrasib received FDA approval for the treatment of previously treated KRAS NSCLC patients [68].

**Table 1:** Efficacy and safety of treatment of sotorasib and adagrasib in the CodeBreak100 and the KRISTAL-1 trials.

Drug/Dose	Phase	Population	N. of Enrolled Pts	N. of Previous Lines (Median)	Efficacy	Treatment-Related AEs
CodeBreak100 [61] Sotorasib 960 mg daily	Phase I/II	Locally advanced or metastatic NSCLC KRAS G12C mutation as assessed by central testing on tissue Stable brain metastases allowed	126	2	ORR: 37.1% DCR: 80.6% mPFS: 6.8 (5.1–8.2) mOS: 12.5 (10-NE)	G1-4: 69.8% G ≥ 3: 20.6%
KRISTAL-1 [66] Adagrasib 600 mg daily	Phase I/Ib	Unresectable or metastatic stage Treated or stable brain metastastis allowed	116	2	ORR: 42.9 DCR: 79.5 mPFS: 6.5 (4.7–8.4) mOS: 12.6 (9.2–19.2)	G1-4: 97.4% G ≥ 3: 44.8%

ORR: objective response rate; PFS: progression-free survival (months, 95% CI); OS: overall survival (months, 95%CI).

### 2.3. Novel Molecules for Targeting KRAS Mutations

Novel KRAS G12C inhibitors are currently being evaluated in multiple phase I–II clinical trials. 

A new anti-KRAS inhibitor (JNJ-74699157) has been recently tested in a phase I trial. Ten patients, including 5 NSCLC cases, have been enrolled. Due to the high toxicity profile (grade 3–4 increased blood creatinine phosphokinase) and the lack of clinical efficacy, this trial has been prematurely interrupted. Similarly, the phase I evaluating LY3499446 compound has been stopped because of unexpected toxicity [29]. 

The efficacy and safety of GDC-6036 has been recently presented at 2022 WCLC. In the phase I trial evaluating this novel molecule, GDC-6036 was given to 135 patients with advanced solid tumors, including 59 patients affected by KRAS-mutated NSCLC. In the NSCLC cohort, the median age was 67 years, and the majority received prior checkpoint inhibitor therapy (86%) and platinum-based chemotherapy (90%). The median number of prior therapies was 1 (range, 0–5). As for safety, grade treatment-related AEs were observed in 88.1% of all patients, the most common being nausea (76.3%), diarrhea (61%), vomiting (54.2%), fatigue (23.7%), decreased appetite (15.3%), increased alanine aminotransferase (13.6%), increased aspartate aminotransferase (10.2%), and dyspepsia (6.8%). Severe treatment-related AEs were observed in 16.9% of all patients and the most frequent were increased alanine aminotransferase (6.8%), increased aspartate aminotransferase (5.1%), diarrhea (3.4%), and fatigue (1.7%). No dose-limiting toxicities were reported. As for efficacy, a confirmed ORR was reported in 46% of cases [69,70]. 

Other anti-KRAS G12C inhibitors are currently being evaluated, including PJ34, a molecule that modifies the nuclear mitotic apparatus (NuMA). In NSCLC cell lines, this molecule efficiently determined cancer cell death via an exclusive structural faults inserted in their mitotic spindle. Due to a more selective mechanism of action, PJ34 eventually spared healthy NSCLC cells, with a potentially better tolerance in clinic [71]. However, no ongoing clinical trial is currently evaluating the efficacy and safety of PJ34. Other KRAS inhibitors in ongoing trials are listed in Table 2, with no results posted to date [72,73,74,75,76,77,78]. Of note, these novel compounds will also be tested in patients who progressed after previous KRAS G12C inhibitors, and combinational strategies with other molecular targets implied in resistance will also be evaluated (see moreover).

Molecular targeting of KRAS mutations other than G12C still represents a pioneer domain. KRAS G12D inhbitors (e.g., MRTX1133 and JAB-22000) as well as KRAS G12V molecules (e.g., JAB23000) are currently being investigated with promising preclinical results [79,80]. However, no clinical data are available to date.

## 3. Resistance Mechanisms to KRAS Inhibitors 

Despite these encouraging results, patients exposed to KRAS inhibitors may further progress. Two different mechanisms of acquired resistance to KRAS inhibitors have been identified to date: on-target and off-target mechanisms. On-target mechanism of resistance typically involves the switch pocket II, thus impairing drug binding. In KRAS-mutated NSCLC cell lines, patterns of on-target acquired mutation varied after exposure of either sotorasib or adagrasib. Indeed, KRAS G13D was the most frequently observed on-target mutation in sotorasib-resistant clones (23%), while KRAS Q99L represented the most common mutation after adagrasib exposure (52.8%). Of note, sotorasib-and adagrasib-resistant clones were both associated with Y96D and A59S point mutations [31]. Importantly, a new KRAS inhibitor RM-018 proved preclinical evidence of efficacy in overcoming secondary acquired KRAS G12C/Y96D mutations [31,81], although these data need to be validated in prospective clinical trials. Other KRAS point mutations identified to date in pre-clinical setting include R68S/M, H95D/Q/R and Y96C. Importantly, cross-resistance to sotorasib and adagrasib have been demonstrated for Y96C and Y96D mutants. At the opposite, G13D and A59S secondary mutations remained partially sensitive to adagrasib, while Q99L and H95D/Q/R gathered responsiveness to sotorasib [31]. In clinic, reported cases of acquired G12D/R/V/W, G13D, Q61H, R68S, H95D/Q/R, and Y96C point mutations have been described after progression to adagrasib, while patients progressing after sotorasib further develop G12D/V/F, V8L point mutations [82,83]. 

Multiple off-target mechanisms of resistance have been identified to date and include: bypass signaling pathways, epithelial-to-mesenchymal transformation (EMT) and transition to senescence. 

Activation of bypass signaling pathways typically involves MET amplifications, activating mutation in NRAS, BRAF, MAP2K1, and RET, fusions of ALK, RET, BRAF, RAF1, and FGFR3 as well as loss-of-function of NF1 and PTEN. Of note, these alterations were equally identified after progression to both sotorasib and adagrasib, and were also detected in cell-free DNA [81,82,83].

Epithelial-to-mesenchymal transformation (EMT) has also been reported as mechanisms of resistance both in preclinical and in clinical settings. In sotorasib resistant clones, EMT was associated with E-cadherin downregulation and vimentin upregulation, while patients progressing after adagrasib experienced transition from adenocarcinoma to squamous cell carcinoma histology [83]. Of note, RTK activation (and particularly IGFR1 and FGFR) has been reported in sotorasib resistant cells which displayed EMT [81].

Finally, activation of the aurora kinase signaling could represent a key element explaining transition to senescence as acquired mechanism of resistance. As such, ongoing trials are currently evaluating the efficacy of anti-aurora kinases combined to novel KRAS inhibitors after progression to either sotorasib or adagrasib (see above) [81]. 

Although significant progresses in this field have been made, mechanisms of resistance to KRAS inhibitors are to date only barely understood. Importantly, a putative mechanism of resistance to adagrasib has been found in only about 45% of cases, while 41% of them displaying more than one molecular alteration [83]. With the aim of improving the knowledge in this field, the ongoing SPARK trial is currently assessing molecular alterations detected via ctDNA after progression to KRAS inhibitors [84]. 

Another key issue is how to treat patients after progression, since subsequent lines of therapy could strongly depend on the mechanism of resistance identified at progression. A possible scenario could be adding KRAS inhibitors to targeted compounds depending on the acquired mechanism or resistance. Of note, preliminary results obtained from the phase Ib-II CodeBreak 101 trial demonstrated the efficacy of sotorasib combined with either afatinib or the SHP-2 inhibitor RMC-4630 in patients progressing after KRAS inhibitors, thus highlighting a subset of patients who could benefit from this combined approach at progression to KRAS inhibitors [85,86]. 

## 4. Immune Checkpoint Inhibitors (ICI) in KRAS Mutant NSCLC

Efficacy of ICIs in KRAS mutant patients still needs to be fully assessed. 

Biologically, KRAS mutations have been associated with a pro-inflammatory tumor microenvironment mainly via higher PD-1/PD-L1 expression, CD8+ tumor-infiltrating lymphocytes (TILs) and TMB [87,88,89], and other data also demonstrated that PD-L1 expression is higher in KRAS mutant versus wild-type cases (PD-L1 in KRAS mutant vs. wild-type: 65.3% vs. 58.5%, *p* = 0.0002) [90]. However, discrepancies have been reported, with some meta-analysis rather disconfirming a significant association between PD-L1 status and KRAS mutations [91,92]. 

Consistently with these contrasting results, the clinical outcomes of immunotherapy-treated KRAS mutant patients are equivocal.

In an exploratory analysis of the Keynote-042 trial, KRAS mutant patients benefited from first-line pembrolizumab [93], and a further cohort study also confirmed a longer OS in KRAS mutant patients compared with wild-type after ICI exposure (mOS KRAS mutants vs. wild-type: 21.1 vs. 13.6 months, *p* = 0.03) [94]. In strike contrast, a multicentric retrospective study found no significant differences in terms of ORR, PFS, and OS between KRAS mutants versus wild-type patients (ORR KRAS mutants vs. wild-type: 18.7 vs. 14.4%, *p* = 0.348; mPFS KRAS mutants vs. wild-type; 3.09 vs. 2.66 months, *p* = 0.584; OS KRAS mutants vs. wild-type: 14.29 vs. 11.14 months, *p* = 0.682) [95], and both KRAS mutant and wild-type equally benefited from atezolizumab in the subgroup analysis of the OAK trial [96]. Likewise, discordant results were also observed in three meta-analyses evaluating the efficacy of ICI in KRAS mutant NSCLC patients [97,98,99,100]. 

Compared to other oncogenic drivers, KRAS mutants have been associated with higher responses to ICIs. In the IMMUNOTARGET registry, KRAS positive patients experienced the highest ORR (26%) and long term responses were frequently observed in KRAS mutant cases compared to those with other molecular drivers (12 months PFS KRAS: 25.6%, MET 23.4%, BRAF 18.0%, EGFR 6.4%, ALK 5.9%, HER2 13.6%, and RET 7.0%). Of note, KRAS mutants had the lower rate of rapid progression to ICI (<2 months) compared to those harboring other oncogenic alterations (KRAS 36%, EGFR 44.8%, ALK 45.5%, ROS1 42.9%, RET 43.8%) [101].

Given these contradictory results, it has been wondered if the efficacy of both immune checkpoint and KRAS inhibitors could be boosted up by adding these two molecular classes. So far, multiple ongoing trials are currently evaluating this treatment strategy without any reported data [102,103]. Encouraging results have been recently presented for the CodeBreak 100/101 phase Ib trial. In this trial, adding either pembrolizumab or atezolizumab to sotorasib resulted in a confirmed response of 39% (17/58 patients; range 0–67), and responders had a mDOR of 17.9 months (range 1.5+–23.4). For all patients, mOS was 15.7 months. Importantly, treatment-related AEs were observed in 79–100% of patients and hepatotoxicity was the most frequently observed grade 3–4 AE (30–50%) [104]. Other ongoing trials are evaluating this combinational strategy, without any reported results to date.

As already mentioned, differences in clinical and pathological characteristics have been identified across single KRAS mutant variants. Thus, it has been wondered if the immune infiltrate could also vary depending on KRAS point mutations. 

In preclinical data, KRAS G12D mutants have been linked to a “cold” immune phenotype mainly via PD-L1 downregulation, reduced secretion of pro-inflammatory chemokines (CXCL10/CXCL11), and decreased CD8+ TILs [105]. Furthermore, a lower level of PD-L1 expression and tumor mutation burden (TMB) in KRAS G12D has also emerged in a more recent study (PD-L1 tumor proportion score KRAS G12D vs. non G-12D: 1% vs. 5%, *p* < 0.01; TMB G12D vs. non-G12D: median 8.4 versus 9.9 mt/Mb, *p* < 0.0001) [106]. Again, these data are not univocal and other studies denied any difference in PD-L1 expression across KRAS mutational subgroups [90]. In the aforementioned IMMUNOTARGET registry, no differences in terms of PFS were observed when comparing KRAS G12C to other KRAS mutations or KRAS G12D to other KRAS mutations (*p* = 0.47) or G12D (*p* = 0.40) and similar outcomes were observed when comparing KRAS G12D, G13D, and G12S versus KRAS G12C, G12A, G12V, and G13C mutations [101]. Similarly, other retrospective data excluded any influence on treatment outcomes across single mutant KRAS variants [107]. Conversely, other data highlighted worse treatment outcomes for KRAS G12D compared to non-G12D in those receiving single agent ICI but not after chemo-immunotherapy combinations [106].

It has been supposed that the co-mutational pattern could play a role in pro-immunogenic infiltrate. Indeed, lower levels of PD-L1 expression, TILs infiltration, and higher rates of T-cell exhaustion have been reported for concomitant STK11/KEAP1 co-mutations, thus underlying a subset of KRAS patients that could not benefice from immunotherapy [76,77,78,79]. On the contrary, concomitant KRAS/TP53 mutants have been associated with an increased expression of PD-L1 and a higher mutational load, as well as with a decreased expression of non–PD-L1 immune inhibitory checkpoints, such as Lymphocyte Activating 3 (LAG3) and V-Set Domain Containing T Cell Activation Inhibitor 1 (VTCN1) [108,109]. 

Clinical evidence also supported these data. As such, patients with concurrent alterations in either STK11 or KEAP1 had a significantly worse response to ICI treatment compared to those without these concomitant alterations [110,111,112]. Similarly, patients with TP53 mutations experienced better outcomes from ICI treatment than those harboring TP53 wild-type in multiple series [108,113,114]. 

## 5. Discussion

Sotorasib and adagrasib represents the firsts anti KRAS molecules entered in clinical trials and receiving FDA approval for the treatment of KRAS-positive NSCLC patients in subsequent lines. 

Although these two drugs have been evaluated in similar subsets of patients, noteworthy differences in terms of toxicities emerged so far. As such, all grade treatment-related AEs experienced with sotorasib were significantly lower than adagrasib (all grade treatment-related AEs sotorasib: 69.8%; all grade treatment-related AEs adagrasib: 97.4%), and two cases of grade 5 treatment-related AEs have been reported in the KRYSTAL-1 trial, while no fatal event was described among sotorasib treated patients. The reasons underlying this supposed discrepancy in the toxicity profile need to be fully understood and further validated. Importantly, efficacy was similar among both these molecules, and sotorasib recently confirmed its superiority over docetaxel in the phase III CodeBreak 200 trial [63]. 

Despite these encouraging results, however, responses observed are at around 40% of cases, which is considerably lower than that observed with other oncogenic drivers. As such, two questions may arise: Which would be the reasons behind this suspected lower efficacy? How can it be improved? 

Regarding the first question, we need to remember that KRAS is only one of the three RAS isoforms that normally co-exist in human tissues and that its downstream pathway could be activated via multiple bypass cascades. Therefore, the blockage of KRAS mutants could be easily bypassed via other wild-type RAS isoforms or further escape pathways, such as ERBB family members (see moreover). Furthermore, Passaro A. pointed out that the mechanism of action of KRAS inhibitors significantly differs from that observed for other TKI inhibitors, since it blocks KRAS mutants in their GDP inactive form [115]. Whether and how this difference in the mechanism of action could impact drug effectiveness still needs to be fully understood. Moreover, besides KEAP1 and STK11, little is known about the predictive role of other concomitant mutants and the real impact of concomitant mutations other than STK11/KEAP1/TP53 in determining KRAS inhibitor’s efficacy still needs to be assessed. Additionally, it is still an open question to what extent patients with other oncogenic drivers (e.g., EGFR, METex14, ALK, ROS1) who develop KRAS mutations as the mechanism of escape could effectively benefit from KRAS inhibitors. 

Regarding the second question, multiple ongoing trials are currently evaluating the efficacy of combining sotorasib or adagrasib with other agents (Table 3). One potential combination strategy includes molecules that target either the upstream or downstream signaling pathway. 

As mentioned above, KRAS activation may involve other proteins besides Grb2 and SOS-1, such as the ubiquitously expressed phosphatase SHP-2. The role of SHP-2 in activating the KRAS pathway is still controversial but evidence suggests that it could serve as a scaffold protein to Grb2/SOS1 after RTK phosphorylation, thus aiding in KRAS upstream transduction signaling [9]. Furthermore, SHP-2 inhibition resulted in a higher cell senescence and lower grade of tumor development in preclinical models of KRAS mutant NSCLC [120,121], with a synergistic effect observed when combining adagrasib with the anti-SHP-2 agent RMC-4550 [59]. 

Primary results of the ongoing phase Ib-II multiple cohorts CodeBreaK 101 trial were recently presented. In this trial, patients with advanced solid tumors harboring KRAS G12C received sotorasib either alone or combined with other molecules, including the SHP-2 inhibitor RMC-4630 [116]. The primary endpoint was safety and tolerability, key secondary endpoints included ORR and pharmacokinetics. Results from the cohort of patients treated with sotorasib and the SHP-2 inhibitor RMC-4630 have been recently presented. Among the 11 NSCLC patients included, 3 (27%) had PR and 7 (64%) has disease control and this percentage was higher among KRAS inhibitor naïve patients (75% PR and 100% disease control). The safety profile was manageable, and main AEs consisted of diarrhea and transaminase increase [85]. Other ongoing trials are evaluating the efficacy of anti-SHP2 or anti SOS-1 molecules, with no data reported yet [117,119,122].

Another potential strategy in targeting the upstream pathway could rely on the inhibition of EGFR. In contrast with the previous thought of KRAS mutant independency from upstream RTK, recent data demonstrate that the genetic abrogation of EGFR resulted in impaired tumor growth of KRAS mutant NSCLC models. Of note, EGFR inhibition was rapidly overcome via alternative pathways that include the activation and overexpression of other ERBB family members [123]. On thisasis, it has been supposed that co-targeting KRAS with a pan-ERRB inhibitor such as afatinib could potentiate KRAS-inhibitors’ efficacy. Indeed, preclinical evidence pointed out a significant reduction of tumor growth when afatinib was combined with anti-KRAS molecules [59]. 

The combination of sotorasib and afatinib is currently being investigated in the aforementioned CodeBreak 101 trial. Firsts results were presented at AACR in 2021. Afatinib was given in two different scheduled (either 20 mg or 30 mg daily, cohort 1 and cohort 2 respectively) and combined to sotorasib 960 mg daily. A total of 33 NSCLC patients were enrolled, of whom 10 patients were in cohort 1 (afatinib 20 mg + sotorasib 960 mg) and 23 patients in cohort 2 (afatinib 30 mg + sotorasib 960 mg). In both cohorts, patients received a median number of two prior therapies (range 0–7; 66.7% ≥ 2 prior lines) and five patients (15.2%) received prior sotorasib. The combination of sotorasib and afatinib resulted in an ORR of 20% (cohort 1) and 34.8% (cohort 2), DCR was also higher in cohort 2 than cohort 1 (DCR 70.0% in cohort 1, DCR 73.9% in cohort 2). In terms of toxicity, all grade treatment-related AEs were mainly gastrointestinal (diarrhea 69.7%, nausea 21.2%, vomiting 18.2%). Grade ≥3 treatment-related AEs were reported in 30% of patients in each cohort, with diarrhea being the most frequent (21.2%). Eight patients (24.2%) discontinued sotorasib and/or afatinib due to an AE [86]. Other trials are still ongoing and will further assess the efficacy and safety of KRAS inhibitors combined with EGFR/panERBB inhibitors [102]. 

As what concerns downstream pathways, preclinical experiments demonstrated that KRAS mutants are associated with higher ERK1/2 phosphorylation and that sensitiveness to MEK inhibitor is higher for G12C than for G12D mutants [124]. Furthermore, PI3K can directly be activated by RAS proteins, thus representing a direct RAS effector [125]. As such, the combination with anti-KRAS and inhibitors that insist on RAF/MEK or PI3K/AKT pathway have been tested in preclinical settings, with promising data [59,126]. Multiple ongoing trials are currently assessing the efficacy and safety of combination treatment with sotorasib/adagrasib and molecules which either inhibit PI3K/ATK/mTOR or RAF/MEK pathways, with no reported data available [116,118]. 

Preclinical data demonstrated that KRAS mutations might induce chemo and radio-resistance via multiple mechanisms, such as the induction of senescence, imbalance in ROS production, and activation of mechanisms of DNA repair [127]. Importantly, resistance to radiation has also been demonstrated in some clinical reports [81]. As such, blocking KRAS mutations could eventually reverse radiation resistance, with obvious positive consequences. Clinical trials evaluating radiation combined with sotorasib are ongoing and would further elucidate this potential approach [128,129]. 

Finally, other open questions remain, such as which would be the best timing for treatment with KRAS inhibitors and which would be the best therapeutic option in those who progressed to KRAS inhibitors. In this context, ongoing trials will further elucidate the effectiveness of KRAS inhibitors in neoadjuvant, locally advanced, and first line settings [103,129,130,131,132,133]. 

In conclusion, KRAS mutations in NSCLC represent a topic of growing interest and, not surprisingly, literature reviews on this subject have exponentially increased over time. Nevertheless, we think that our work could be of added value for multiple reasons. First, it represents a very up-to-date article about the clinical efficacy of KRAS inhibitors, including the most recent trials results. This is particularly remarkable if we consider that the last review on this topic dates back to August 2022 and that the treatment scenario rapidly evolved since then [134]. Secondarily, many previous reports alternatively focused on specific aspects within this topic, such as mechanisms of resistance rather than therapeutic agents’ efficacy [135,136,137], leaving behind some other important factors that should have been conversely considered. Nevertheless, two recent reviews may display some similarities with our paper. In their paper, Huang et al. also described the heterogeneity of clinical characteristics in KRAS mutants, co-mutant alterations, immature clinical data from KRAS inhibitors, as well as combinational strategies. Nonetheless, their paper has a rather preclinical approach and barely mentioned novel KRAS inhibitors such as GCD-6036, while completely missing clinical data on the efficacy of immune checkpoint inhibitor’s in KRAS mutant patients [81]. In contrast, Kwan et al. published a clinically focused article on KRAS G12C inhibitors and clinical data from novel KRAS inhibitors as well as combinational strategies and these were brilliantly reported [29]. Strikingly, no reference to either co-mutations or clinical heterogeneity across KRAS mutant patients has been described. Of note, none of these two aforementioned papers referred to some aspects that were conversely described in our review, such as the prognostic and predictive significance of KRAS mutations and diagnostic tools for detecting KRAS mutations. 

With the aim of presenting an updated clinical overview on the state of the art of KRAS mutations in NSCLC, we think that our review extensively summarized the main hot topics connected with this subject and could thus represent a resource of added value. 

## 6. Conclusions

Sotorasib and adagrasib are the first KRAS inhibitors that received regulatory approval for the treatment of KRAS G12C mutant NSCLC patients in subsequent settings. Evidence supporting these approvals mainly derive from phase I/II trials, while sotorasib recently demonstrated its superiority over docetaxel in the phase III CodeBreak 200 trial. Despite these data, the efficacy of these new compounds is lower than that observed for other molecular drivers. Furthermore, the efficacy of ICIs in this subpopulation is not yet completely established and could strongly be influenced by concomitant alterations as well as single KRAS mutational variants. Open issues concerning the increase of efficacy, the definition of mechanisms of resistance, and the best timing for treatment with anti-KRAS molecules are currently being assessed in ongoing trials. 

## Figures and Tables

**Table 2:** Ongoing trials evaluating novel inhibitors for KRAS-G12 mutant advanced NSCLC.

Trial	Phase	Setting	Stage	Pts	Treatment	Primary Endpoints
NCT04449874 [70]	Phase I	Naive or pretreated	Stage IV	498	GDC-6036 (KRAS G12C inhibitor) +/− other molecules: Atezolizumab GDC-1971 (SHP2 inhibitor) Bevacizumab Cetuximab Erlotinib	Safety
NCT04699188 [73]	Phase Ib/II	Pretreated	Unresectable/Metastatic	425	JDQ-443 (KRAS G12C inhibitor) + other molecules: TNO155 (SHP2 inhibitor) Tislelizumab (anti-PD1)	Dose esclataion: DLT, safety and tolerability, dose intensity by treatement Dose expansion: ORR
NCT04956640 [78]	Phase Ia/Ib	Pretreated (also after G12C inhibitors)	Locally advanced, unresectable, metastatic	360	LY3537982 (KRAS G12C inhibitor) + other molecules: Abemaciclib Erlotinib Pembrolizumab Temuterkib (ERK inhibitor) Cetuximab LY3295668 (Aurora A inhibitor) TNO155	Phase 1a: RP2D, DLT Phase 1b: safety and tolerability
NCT04973163 [75]	Phase Ia/Ib	Pretreated	Locally advanced, metastatic	72	BI 1823911 (KRAS G12C inhibitor)+/− BI 1701963 (SOS1 inhibitor)	Dose escalation: DLT Dose confirmation and expansion: OR (BOR of confirmed CR or PR)
NCT05288205 [74]	Phase I/IIa	Preatreated	Locally advanced, metastatic	124	JAB-21822 (KRAS G12C inhibitor) + JAB-3312 (SHP2 Inhibitor)	RP2D, MTD, safety
NCT05379946 [76]	Phase I/II	Preatreated	Locally advanced, unresectable, metastatic	92	D-1553 (KRAS G12C inhibitor) + IN10018 (FAK inhibitor)	Safety, ORR
NCT05067283 [77]	Phase I	Preatreated	Unresectable, metastatic	264	MK-1084 (KRAS G12C inhibitor) +/− pembrolizumab	DLT, safety

DLT: dose-limiting toxicity; ORR: objective response rate; R2PD: recommended phase 2 dose; OR: objective response; BOR: best objective response; CR: complete response; PR: partial response.

**Table 3:** Ongoing trials evaluating sotorasib/adagrasib combined with other agents in KRAS-G12 mutant advanced NSCLC.

Trial	Phase	Setting	Stage	Pts	Treatment	Primary Endpoints
NCT04185883 [116] (CodeBreak 101)	Phase Ib/II	Pretreated, naïve from KRAS G12C ihibitors	Stage IV	1054	Sotorasib +/− other molecules AMG-404 (anti-PD1) Atezolizumab Pembrolizumab Afatinib Trametinib RMC-4630 (SHP2 inhibitor) Everolimus Palbociclib TNO155 (SHP2 inhibitor) Carboplatin, pemetrexed, docetaxel	Phase Ib: Cmax, Tmax, AUC, ORR, DCR, DoR, PFS, Duration of Stable Disease, TTR, OS Phase II: safety, Cmax, Tmax, AUC, DCR, DoR, TTR, OS
NCT03785249 [102] (KRYSTAL-1)	Phase I/II	Naive or pretreated	Stage IV	740	Adagrasib +/− other molecules: Pembrolizumab Afatinib Cetuximab	Safety, blood plasma concetration, ORR
NCT04613596 [103] (KRYSTAL-7)	Phase II	Treatment naïve	Unresectable/metastatic	250	Adagrasib +/− pembrolizumab	ORR
NCT04330664 [117] (KRYSTAL-2)	Phase I/II	Naïve or pretreated	Unresectable/metastatic	86	Adagrasib + TNO155 (SHP2 inhibitor)	Safety, blood plasma concentration
NCT05074810 [118]	Phase I/II	Pretreated	Stage IIIB-IV	53	Sotorasib + VS-6766 (RAF/MEK inhibitor)	PartA: RP2D, DLTs Part B: ORR
NCT04975256 [119]	Phase I/Ib	Naïve or pretreated	Unresectable/metastatic	100	Adagrasib + BI 1701963 (SOS1 inhibitor)	Safety, blood plasma concentration, DLT

Cmax: Maximum Plasma Concentration, Tmax: Time to Maximum Plasma Concentration, ORR: objective response rate, DCR: disease control rate, PFS: progression-free survival, DoR: duration of response, TTR: time to response, OS: overall survival; R2PD: recommended phase 2 dose; DLT: dot-limiting toxicities; OR: objective response; CR: complete response; PR: partial response.
